# The research program subjective theories: A methodological concept for a wide range of applications

**DOI:** 10.3205/zma001799

**Published:** 2026-01-15

**Authors:** Pia Natalie Gadewoltz

**Affiliations:** 1 Bielefeld University, Medical School OWL, Department for University Study and Teaching, Bielefeld, Germany; 2 Osnabrück University, Institute of Health Research and Education (IGB), School of Human Sciences, Osnabrück, Germany

**Keywords:** subjective theories, communicative validation, dialogue-consensus, structure-formation-techniques, qualitative research, interview

## Abstract

The article introduces the “research program subjective theories” (RPST), which was developed in the 1980s to systematically investigate individual thought processes. The focus lies on “subjective theories” – stable, structured systems of convictions that function in the same way as scientific theories, but are less formalized. Central to the RPST is the dialogical reconstruction of such theories through the exchange between researchers and interviewees, which leads to authentic insights and reflection of thought processes. The “structure-formation-technique” (SFT) method reconstructs and visualizes these processes. The RPST has evolved over the years and is now used in a variety of ways by research and education, and also expands the diversity of scientific approaches in medical education research.

The article summarizes the scientific-theoretical fundamentals and research-methodological background of RPST and provides an overview that can be used as a basis for taking the first steps toward theory-based application of this approach.

## Abstract (de)

Der Artikel stellt das „Forschungsprogramm Subjektive Theorien“ (FST) vor, das in den 1980er Jahren entwickelt wurde, um individuelle Denkprozesse systematisch zu erforschen. Im Fokus stehen „Subjektive Theorien“ – stabile, strukturierte Überzeugungssysteme, die wie wissenschaftliche Theorien funktionieren, aber weniger formalisiert sind. Zentral im FST ist die dialogische Rekonstruktion solcher Theorien durch den Austausch zwischen Forschenden und Befragten, was zu authentischen Einblicken und Reflexion der Denkprozesse führt. Die Methode der „Struktur-Lege-Sitzung“ rekonstruiert und visualisiert diese Prozesse. Das FST hat sich über die Jahre weiterentwickelt, wird heute von der Forschung bis zur Bildung vielfältig genutzt und erweitert auch die Vielfalt der Forschungsansätze in der medizinischen Ausbildungsforschung.

Der Artikel fokussiert in einer verdichteten Form die wissenschaftstheoretischen Grundlagen und forschungsmethodischen Hintergründe des FST und bietet auf dieser Basis einen Überblick für die ersten Schritte zur theoriebasierten Anwendung der Forschungsmethodik.

## 1. The research program subjective theories

In 1988, Groeben et al. introduced the “research program subjective theories” (RPST), which was developed in the German-speaking psychology and education research. On the one hand, the RPST describes specific, structuring concepts, and on the other hand, it integrates a research methodology based on key concepts. The RPST represents an appropriate method for medical education research, as it enables a well-founded analysis and evaluation of the subject-oriented view of teaching and learning processes. This article provides an introductory overview (for a detailed description of the method in the context of interprofessional teaching, see [1]).

The RPST is primarily applied in educational research, but also in psychological basic research, in areas of pedagogical and clinical psychology, as well as in adjacent scientific disciplines [2], such as economics, health sciences, technology research, and also in research on medical education and other health-related professions [3], [4], [5], [6], [7], [8], [9]. The potential for the application-related use of the RPST is demonstrated by the example of interprofessional education (see chapter 4). The RPST is thus a versatile tool for investigating educational processes. Furthermore, the methodology offers the possibility of providing valuable impulses to the researched field and supporting reflective, didactic, and evaluative processes (see chapter 5).

The methodology of the RPST allows for the reconstruction of individual conceptions and action-guiding thought processes as so-called subjective theories [3]. The participants are considered as mature and equal individuals in the research process, who are attributed the ability to engage in rational and goal-directed actions that can be named and explained in communicative processes [4]. The prerequisite for this “epistemological model of the human being” [3] is a cooperative collaboration of all parties involved.

“Subjective theories” (ST) are complex, relatively stable, and enduring cognitions about the self and the worldview. They are highly individual and exhibit at least an implicit, argumentative structure. They also have explanatory and/or predictive functions for the subject, ranging from general orientation, explanation of past experiences, or prediction of future events to complex action control [10]. ST are usually rational and verbalizable and exhibit a significantly higher degree of consistency and accessibility of cognitions compared to convictions or “beliefs” [11]. Convictions are based on one’s own evaluations and assessments [12], but do not necessarily have to be consistent within themselves, and can even be inconsistent with actual behavior [13]. ST are understood both in their structure and function as analogous to scientific theories, but they lack the intersubjectivity and explicitness of the latter. In comparison to scientific theories, the coherence of ST is limited, as they must enable quick reactions under certain circumstances, but do not need to be the subject of extensive discussions or evaluations [10].

The RPST assumes that people are generally capable of reflecting on themselves and their environment and that they can arrive at action-guiding insights [2], [10]. A classification of ST refers to their scope of influence, according to the extent of their impact. ST with a narrower scope are characterized by conceptions of concrete action sequences, such as concrete reactions to the behavior of individuals or groups [2], [14]. The individual, cognitive constructs refer to processes and structures that explain, control, or guide actions. For ST with a medium and large scope, the conceptions no longer refer to the specific action sequences, but are related to more comprehensive theoretical concepts, such as hypotheses and argumentation structures on a higher level of abstraction. The ability to engage reflexively, in a structure-parallel manner to scientific theorizing, with one's own cognition in an explanatory and action-guiding way leads to the anthropological assumption that underlies the RPST and its research methodology.

## 2. The foundation: Basic assumptions and methodology of the research program subjective theories

### 2.1. Basic assumptions and basic concepts of the RPST

A human model that is characterized by “intentionality, reflexivity, potential rationality, and linguistic communicative ability” and therefore considers humans as “acting subjects” [15] is fundamental to the RPST. According to this, one’s own actions are not only thought through in terms of goals, justifications, and structures, but can also be communicated. The persons to be researched (cognitive objects) possess a communicable action and decision-making ability that is used for research purposes, but also has an impact on the research process itself [15]. For the RPST, it is fundamental that the human being as an acting subject is at the center of consideration. Furthermore, the distinction between the concepts “behavior” and “action” is important. From the perspective of behaviorism, behavior can occur without reflection, with people being influenced by environmental stimuli [3]. In contrast, action is goal-oriented and intentional, characterized by the core feature of intentionality, and is considered a sub-level of behavior [16], [17]. The thought processes underlying actions are not immediately visible from the outside, but can be made visible through verbal communication and exchange about them. This allows for the comparison of the understanding of the researchers with that of the researched, which is described in the RPST as “dialogical consensus” [3]. The mutual dependence of research object and research methodology was of great importance for the development of the RPST [18], based on the assumption that research does not have an unobstructed, neutral possibility of accessing its research object. The latter can only be represented within the scope of the options and with the limitations of the selected method. To take this into account, the RPST was developed as a specific methodological approach, which this article introduces in an orienting and introductory manner.

### 2.2. The subject of ST

According to Groeben et al., the external perspective of the observer and not the self-perspective of the acting subject is represented when researchers alone try to access the subjective theories of the cognitive object, for example, through the interpretation of an interview [10]. The essential characteristics of the epistemological model of the human being would not be utilized in this case. If the exchange with the cognitive object is left out, Groeben et al. speak of a “monologue hermeneutic” approach that only allows for a descriptive understanding of the researchers [10]. In contrast, the researched cognitive objects can provide information on the meaning of the relevant action as well as the internal conditions and starting points. This self-reporting includes the underlying human model as an acting subject and enables access to the action effectiveness [10].

### 2.3. Procedure in the research process of the RPST

#### 2.3.1. Communicative validation 1 – collection and preparation of cognitive content

The research methodology of the RPST, as described, has a specific focus on the dialogue with the participants. Validation therefore does not only serve to check on the consistency and comprehensibility of the results in the later research process, but is implemented as communicative validation already in the process of data collection.

The RPST does not assume that cognitive objects are able to provide an immediate and trouble-free comprehensive and usable description of the reflected inner perspective of their actions. However, it is a particular concern and programmatic claim of the RPST to depict the ability to reflexivity, rationality, and expression of the cognitive objects, as well as to actively involve them. To ensure that the researchers understand and classify the descriptions of the inner perspective appropriately, the reconstruction of the ST is carried out in dialogue, i.e., in a shared argumentative approach. This form of dialogue is referred to as “communicative validation” [10]. In the reconstruction of the ST, own formulations of the cognitive object are used, and the researchers adapt to the language use of the researched. The participants exert influence on the research process and evaluate whether the formulated inner perspective is adequately represented. The researchers thus obtain a descriptive construct of the ST that is accessible to scientific evaluation. The “dialogue consensus criterion of truth” is ensured by the RPST, as the statement of the cognitive object is given greater weight to ensure that the reconstruction result actually corresponds to its inner perspective [10], if researchers and researched do not agree.

Communicative validation ensures that the internal constructs and thought processes of the person being investigated are correctly understood and formulated by the researchers in the dialogue-consensus. It is only in a second phase that it becomes apparent whether the ST also provides a valid explanation of actions and psychological phenomena: with the “explanatory validation”, the reconstructed, descriptive constructs of the ST are checked [3], [10]. Groeben et al. speak of the “adequacy of understanding” of the construct, which is examined in a research phase that is subsequent but superior in terms of content [10]. The observation of the behavior of the person being investigated by the researchers as external persons is intended to secure the ST intersubjectively.

In the first phase of the RPST, the ST is therefore initially “dialogue-hermeneutically” collected, in order to then verify them using the “dialogue-consensus procedure” with the inner perspective of the cognitive object (communicative validation) and to describe the constructs [10]. To avoid overburdening the participants, two steps are taken here: first, the cognitive content of the research object is collected in an interview. To meet both the research questions and subjects of the researchers and the cognitive structures of the interviewed person, semi-standardized interviews are used as a method [3]. The underlying interview guide is supposed to be flexible according to the RPST, so that a natural conversation flow can arise in the interview and the sequence and wording can be adapted to the person being investigated. Subsequently, the interviews are evaluated by the researchers, for example, through content analysis, and the resulting constructs are brought onto cards (construct cards), in order to prepare them for a subsequent structure reconstruction. In addition, cards with formal relationships and references are created (structure cards), which, for example, contain descriptions such as “is equal to”, “is a superordinate concept of”, or “interactions”.

#### 2.3.2. Communicative validation 2 – collection of structural relationships of contents

**Figure 1:**
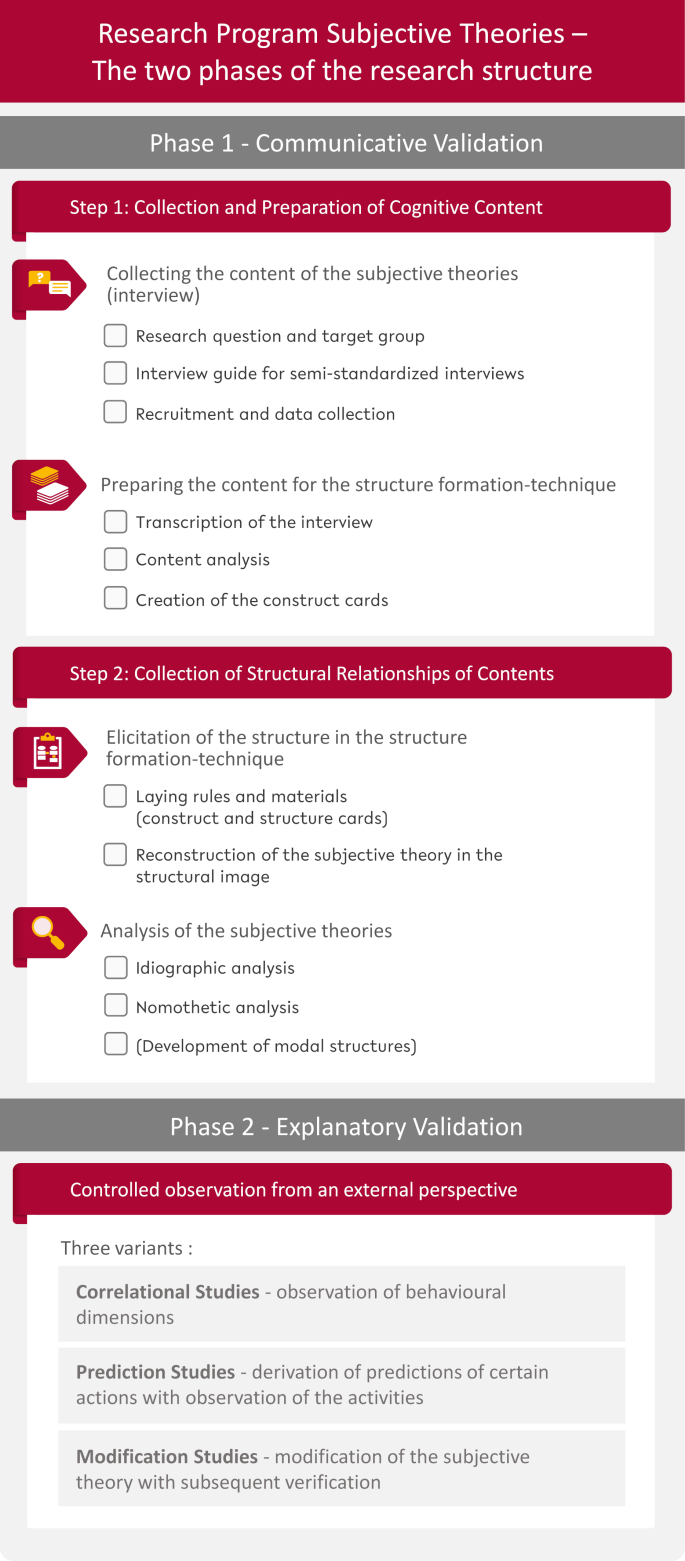
Flowchart of the subjective theories research program (own illustration based on Groeben et al. [10])

The second step with the participant follows. In a structure-formation-technique (SFT) session, a structural image is created using the construct and structure cards, which is then further analyzed [2], [10]. Following the original procedure, the researcher creates a structural image in advance using the cards themselves. This enables a dialogue-consensual approach in the subsequent joint SFT session: a continuous comparison between the cognitions of the participants and the understanding of the researchers takes place, which ultimately leads to a communicatively validated consensus-structural image [3]. A flowchart of the entire process is shown in figure 1 (Fig. 1).

#### 2.3.3. Explanatory validation

The empirical verification of the descriptive constructs and the validation in the form of “explanatory constructs” takes place in the second phase of the methodology of the RPST (explanatory validation) [10]. Observations from the outside perspective are made. Three study variants are described for explanatory validation: correlation, prediction, and modification studies [2], [10]. In correlation studies, elements of the reconstructed ST are related to observational data to correlate inner and outer perspectives. The extent of the correlation is evaluated as an indication of explanatory validity. Prediction studies check the predictive power of the reconstructed ST with regard to the actual behavior of the investigated person and thus what Groeben et al. refer to as “adequacy of understanding” (see above) [10]. In modification studies, modifications of the ST are worked on and the connection between the predicted and actual behavior is investigated in conjunction with the modifications. In this approach, the quality of the prediction is an indication of explanatory validity [10]. The implementation of explanatory validation is methodologically imprecise and, not least due to the high effort it requires, is rarely done [4].

## 3. Methodology in motion: Adaptation and development of the RPST

Through modification and methodological adaptations in the context of research projects, the RPST has been further developed [3], [14]. Various forms of the initially proposed semi-standardized interview have been applied (e.g., the problem-centered interview, the expert interview, or the episodic interview), which differ in their content orientation and formality with regard to the spectrum between strictly guide-based and more narrative interviews [10], [14]. In addition, various categories or formats of questions can be important tools in the context of interviews [10]. In order to make descriptions as realistic and concrete as possible, case studies can be used and/or interviews can be conducted after an event, which can then be referred to [14]. Written surveys of the research subjects can also be conducted in advance to support targeted questions in the subsequent interview [14].

For the preparation of the collected data, two approaches are generally used: on the one hand, the original programmatic approach of the RPST is followed, which involves the analysis and preparation of the contents for a subsequent structure-formation-technique (SFT) session, and on the other hand, the complete reconstruction of the subjective theories by the researchers based on the existing interview material [14]. The second approach contradicts the RPST, as it lacks feedback from the interviewee in the dialogue-consensus process and thus a significant part of their influence. In a program-compliant modification, the interviewee is more closely involved by writing down key terms for the concept cards themselves during the interview [14].

The RPST does not provide detailed methodological information on how researchers extract the “most important concepts” when preparing interview material for concept cards [10]. One option for a rule-based approach is the content analysis adapted to RPST as described by Kindermann [19].

The SFT session, which is conducted according to rules defined a priori by the researchers, should be seen as a central component of the RPST methodology. The approach should correspond to the areas of investigation and the research questions on the one hand, but on the other hand it should also support the research subjects in presenting the content in a structured manner without them having to generate the rules for the approach themselves [3]. Various approaches have been described for this purpose [3], [10], [14], but their introduction would go beyond the scope of this article.

A reduction in the number of concept cards (usually 25-150) or structure cards can simplify the SFT session [14]. The design of the concept cards also offers a range of options, from keywords to half-sentences and examples to whole sentences or statements. In some studies, the subjects also assessed influencing factors using a multi-level scale with a view to inhibiting and promoting factors, respectively. Furthermore, participants may comment on the structure image again, either in part or as a whole, at the end of the SFT session [14]. Following the RPST, the structural image from the SFT session is the only data basis for the final analysis. Current studies often use additional data material, such as comments from the participants or contents from the interviews [14].

The evaluation methods are characterized by qualitative approaches, which are mostly constructive-descriptive in nature. The STs can be analyzed individually (idiographic analysis) or several STs can be subjected to a higher-level consideration (nomothetic analysis). In most cases, individual STs are reconstructed using content-analysis methods. In the higher-level, i. e. nomothetic analysis, the individual representation is abandoned. Contrasts between structural images allow for comparative analyses. Sometimes, groups that differ, for example, in terms of their professional experience, develop summarizing structural analyses known as modal structures [14]. Some of the differences in the collection and analysis of STs arise from the use of different data sources, and different questions may require different approaches [14], but this is perfectly in line with the RPST [3].

## 4. From theory to practice: The many different fields of application for the RPST

The RPST began with basic research in general psychology and social psychology, with research subjects ranging from irony, independence, moral courage, and aggression to identity concepts and identity prognoses [3]. Although the application of the RPST shows a focus on educational science and is primarily anchored in the pedagogical psychology [4], [14], it is important in many other research fields, such as additional areas of psychology, foreign language teaching, economics, psychosomatics, and technology research [3], [4] but also in health sciences, nursing, and therapeutic areas [5], [6], [7], [8], [9].

An overview of research relating to RPST revealed a pronounced orientation towards application, focusing on questions concerning improvements in the actions of professionally acting individuals and their educational, counseling, or medical-therapeutic clients [4]. On the other hand, subjective theories can serve to formulate scientific theories, especially when these are still underdeveloped, but a great deal of empirical knowledge already exists.

Another area of research for which the RPST and its methodology are particularly well suited is interprofessional teaching. Individual thought structures and cognitions influence interprofessional cooperation in practice and also in teaching. What the participants know and think about each other is of great importance when acting professionally together [20], [21]. The knowledge of the underlying, action-guiding structures of thoughts can be used to improve interprofessional cooperation and help to explain existing obstacles. The RPST’s inherent view on individual cognition, combined with the assumption that participants possess (self-)recognition abilities, makes personal perspectives apparent and can, for example, represent (action-)justifications and argumentation structures [10] that can then be used for changes. Important topics for interprofessional teaching are equality and equal rights, which could be supported by a research method that promotes equal rights for the (inter-)acting subjects. Instead of talking about individuals or groups and making decisions for them, actively involving them in the research process would be an approach that promotes interaction “on eye level” between the people involved and can thus serve as an example of equality. In an ideal and safe conversational environment [10], the experiences of the research subjects that are significant to their own ST can contribute to a better understanding of previously unexplained problems or relevant constructs in the research process. Explanatory validation could then confirm whether and to what extent the descriptive constructs hold true in reality, i.e., whether the ST described in the dialogue-consensus procedure really leads to the presumed actions.

## 5. More than just a method: RPST as the key to insight and reflection

RPST was developed as a research method and is predominantly used as such. However, its methodology can be used for more than just generating research results.

Communicative validation within the RPST can serve the development of teaching and education, as the guided reflective process in the SFT session particularly encourages and supports the further development on the levels of thought processes and action decisions. Thus, an effect can occur in the researched field beyond the inherent knowledge-generating approach of the research. This is made possible by the reflexivity of the “acting subject”, which, through the communicative process with the researchers, reflects on the aspects of its own action with goals, justifications, and structures, and can check actions and decisions [15]. As a result, the participants can be motivated by the reflective process to make other decisions or adapt their actions in the further course.

The reconstruction of ST is also useful as a didactic method. The SFT has already been used as a teaching method [22], [23], [24]. In one example, structure formation maps were used for seminars to allow teacher trainees to work out their subjective theories and make them accessible to reflection [23]. In peer discussions, participants used the mutual presentation of their own structural models to enable comparison with the ST of others and to engage intensively with their own thought structures and different concepts. Contents from the structure formation maps could also be used for adaptive adjustment of teaching and thus enable teachers to better align with the actual learning level of their students. Repeated application of the procedure could also be used for evaluation purposes [23]. In summary, it can be said that the research program on subjective theories (RPST) is not only a suitable method for education research, but can also support reflection among the respective target group and improve the quality of the areas under investigation.

## Acknowledgements

I would like to thank Prof. Dr. Ursula Walkenhorst for providing the insightful impulse that prompted my in-depth examination of this research method and its considered application in my research. I also appreciate the encouragement from Prof. Dr. Jan Matthes to write this article.

## Author’s ORCID

Pia Gadewoltz: [0009-0000-0632-6536]

## Competing interests

The author declares that she has no competing interests.
